# Patient-derived induced pluripotent stem cells for precision modelling of monogenic beta cell disorders

**DOI:** 10.3389/fendo.2026.1819394

**Published:** 2026-06-03

**Authors:** Lily Deng, Mansa Krishnamurthy

**Affiliations:** Division of Diabetes and Endocrinology, Cincinnati Children’s Hospital Medical Center, University of Cincinnati College of Medicine, Cincinnati, OH, United States

**Keywords:** beta cell disorders, hyperinsulinism, IPSC, monogenic diabetes, SC-islet

## Abstract

Over the past several years, research and technologies involving patient-derived induced pluripotent stem cells (iPSCs) have rapidly advanced, enabling the study of various disease pathologies, including rare pathologies like monogenic disorders of beta cell function. iPSCs offer a unique model to study monogenic beta cell disorders such as Maturity-onset diabetes of the young (MODY) and congenital hyperinsulinism as they can be derived directly from patients with novel genetic variants and then differentiated into pancreatic progenitors and beta cells. Studies have used iPSC-derived SC-islets to demonstrate how transcription factor dysfunction can perturb insulin secretion, beta cell maturation, and endocrine lineage specification in MODY subtypes. Additional studies in congenital hyperinsulinism have utilized iPSC-derived SC-islets to model disease-specific hallmarks such as excess insulin secretion and increased beta cell proliferation. These studies provide the foundation for future studies using iPSC-derived SC-islets as a platform for elucidating mechanisms of how single-gene variants disrupt beta cell specification, maturation, and survival.

## Introduction

Monogenic disorders of beta cell dysfunction comprise a biologically informative spectrum of syndromes caused by pathogenic variants in single genes that affect beta cell development, insulin secretion, or stimulus secretion coupling. These conditions encompass defects in diverse pathways, including glucose sensing, ion channel function, transcriptional regulation, and hormone processing, and manifest clinically as both insulin deficient states such as neonatal diabetes and maturity-onset diabetes of the young (MODY) and insulin excess states, such as congenital hyperinsulinism (HI) (1–8). Although these conditions are individually rare, monogenic beta cell disorders collectively represent powerful human genetic models that have provided fundamental insight into the molecular regulation of beta cell identity and function across the lifespan. Importantly, precise molecular diagnosis has direct therapeutic implications, enabling genotype guided treatment strategies that differ from conventional approaches to other forms of beta cell dysfunction including type 1 and type 2 diabetes (5, 7). Advances in genomic sequencing are expanding case identification, and studies on monogenic beta cell disorders are increasingly reshaping our understanding of beta cell development and insulin dysregulation.

A major barrier to mechanistic investigation of monogenic disorders of the beta cell has been limited access to primary human pancreas tissue, especially from early developmental time points when many disease phenotypes are established. The advent of induced pluripotent stem cell (iPSC) technology has transformed this landscape. In 2007, it was reported that human somatic cells can be reprogrammed into a state nearly identical to pluripotent stem cells that have the capacity to differentiate into any cell type in the human body (9–11). Initial approaches of generating iPSCs relied on viral transduction of core pluripotency factors (OCT4, SOX2, KLF4, and c-MYC). Advances in subsequent methods have enabled reprogramming using Cre-excisable vectors and non-integrating strategies such as plasmid and episomal transfection (12). These advances have facilitated the generation of iPSC lines that are free of integrated reprogramming factors, improving genomic integrity and the suitability for disease modeling and translational applications.

iPSCs are uniquely well suited for the study of monogenic beta cell disorders. They can be derived directly from patients with novel or pathogenic genetic variants and differentiated into pancreatic progenitors and stem cell-derived islets (SC-islets) (10–12). This platform of study enables patient-specific “disease in a dish” models that recapitulate key aspects of human beta cell development, maturation, and function in a controlled experimental setting. When combined with genome editing to generate isogenic controls, iPSC-based models allow precise dissection of genotype-phenotype relationships (13). Studies using these platforms have demonstrated that monogenic beta cell disorders can reflect defects in insulin secretion as well as perturbations in developmental trajectories, cellular identity, and stress responses, therefore expanding traditional conceptual frameworks of disease pathogenesis (14, 15). However, persistent challenges including incomplete functional maturation of SC-islets and variability across various differentiation protocols demonstrate the need for continued refinement of these models to fully capture adult beta cell physiology (16–18).

In this review, we discuss the application of iPSC-derived SC-islet models to the study of monogenic beta cell disorders with a focus on MODY and HI. We will synthesize findings across studies to identify convergent and divergent mechanisms of disease, including disruptions in transcriptional networks, metabolic signaling, and beta cell excitability. We will also discuss how these platforms are being leveraged to establish genotype-phenotype relationships, interrogate developmental contributions to disease, and enable preclinical evaluation of targeted therapies. Finally, we highlight current limitations and outline future directions, including the need to expand modeling efforts beyond the well-studied K_ATP_ channel disorders, and needed improvements in SC-islet maturation and multicellular complexity.

## Induced pluripotent stem cell-derived islets as a platform for modeling beta cell disease

Collectively, iPSC-based models are a transformative technology with direct implications for mechanistic discovery and personalized treatment in monogenic beta cell disorders. Over the past decade, advances in direct differentiation of iPSCs into stem cell-derived islets (SC-islets) have enabled a scalable and physiologically relevant system for detailed study of the developmental pathways governing human pancreatic beta cell specification, maturation, and function (16, 18). These multistep, developmentally guided protocols recapitulate key stages of pancreatic organogenesis *in vitro*, including definitive endoderm formation, pancreatic progenitor specification, and endocrine differentiation, ultimately producing beta like cells (19–24). Importantly, SC-islet platforms provide a unique platform to model monogenic disorders of the pancreatic beta cell in a human, genotype-specific context. By enabling differentiation of patient-derived iPSCs into pancreatic lineages, these systems allow direct interrogation of how disease-causing variants disrupt lineage allocation, endocrine specification, and beta cell function. Compared to traditional models, iPSC-derived SC-islets capture both developmental and functional dimensions of disease. Despite these advances, challenges persist including variability across differentiation protocols as well as incomplete functional maturation, underscoring the need for continued optimization of SC-islet protocols and systems.

Seminal studies in 2014 demonstrated that SC-islets derived from human embryonic stem cells (hESCs) were capable of glucose-stimulated insulin secretion, establishing a proof-of-concept that functional human beta cells could be generated *in vitro* (21, 22). These foundational studies defined the core framework for directed differentiation. In addition, optimization of key signaling pathways, incorporation of three-dimensional culture systems, and methodological advances of *in vivo* and *in vitro* maturation strategies have significantly improved the functional competence of SC-islets (19, 23, 24). These advances have resulted in enhanced glucose responsiveness, more physiologic insulin secretion dynamics, and transcriptional profiles that resemble mature human beta cells. Despite these improvements, many SC-islet models continue to exhibit some features of functional immaturity, highlighting ongoing challenges in fully recapitulating adult-like beta cell physiology.

**Figure 1:**
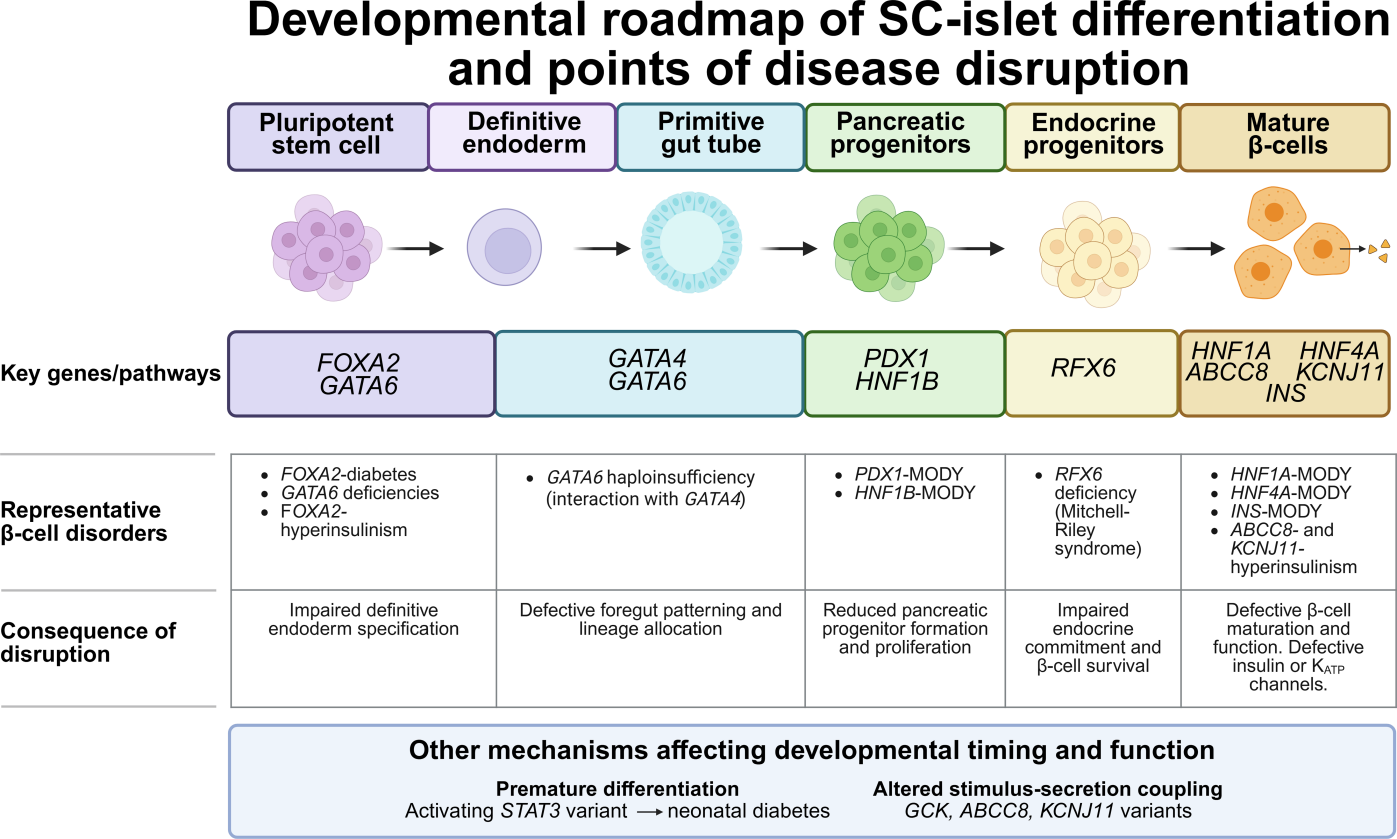
Developmental roadmap of SC-islet differentiation and stage-specific disruption in monogenic beta cell disorders. Human pluripotent stem cells are differentiated through stages that recapitulate human pancreatic development. Key regulators at each stage include FOXA2/GATA6 (endoderm), PDX1/HNF1B (pancreatic progenitor), RFX6 (endocrine commitment) and HNF1A/HNF4A/INS/KCNJ11/ABCC8 (beta cell maturation/function). Representative monogenic disorders are mapped to the developmental stage they disrupt. Additional mechanisms affecting premature differentiation (STAT3) and stimulus secretion coupling (GCK, ABCC8, KCNJ11) are also shown.

Patient-derived SC-islets provide a powerful platform for genotype-specific modeling of monogenic beta cell disorders, enabling the study of defects in beta cell development, insulin synthesis and secretion, and cellular stress responses within a human context. Further, combining this platform of modeling with genetic editing technologies such as CRISPR/Cas9 allows for direct comparison of mutant and isogenic control cell lines. This approach enables the direct comparison between mutant and genetically corrected cells allowing for precise dissection of underlying disease mechanisms, while controlling for genetic background effects. Beyond mechanistic insight, these systems have accelerated translational applications, including the evaluation of targeted therapeutic strategies and pharmacologic screening. Collectively, advances in SC-islet differentiation have deepened our understanding of human beta cell biology and established a scalable platform for precision modeling and the development of cell-based therapies for monogenic disorders of insulin dysregulation. (**Figure 1**).

## Directed differentiation of SC-islets: developmental framework and implications in disease modeling

SC-islet differentiation protocols follow a multi-step, developmentally informed framework that recapitulates key stages of human pancreatic organogenesis (19–21, 23, 24). Human pluripotent stem cells are sequentially directed through definitive endoderm (FOXA2^+^ and SOX17^+^), primitive gut tube, pancreatic endoderm, and endocrine progenitor stages (PDX1^+^ and NKX6.1^+^) before generating hormone-expressing cells. While this progression through development is described as a linear sequence, it is more accurately reflected as a series of tightly regulated cell fate decisions regulated by temporally coordinated signaling inputs, including Activin/Nodal, WNT, retinoic acid, FGF, and TGF-beta pathways. Moreover, each developmental transition represents a point of vulnerability where disease causing variants, such as those associated with MODY or Congenital Hyperinsulinism, can disrupt lineage specification, progenitor competence, or endocrine commitment. Thus, SC-islet differentiation systems not only generate insulin-producing cells but also provide a framework to interrogate how genetic defects impact various developmental stages.

Over the past decade, SC-islet differentiation protocols have evolved with refinements that reflect an increasing understanding of both developmental biology and beta cell physiology. Early landmark studies by Pagliuca *et al*. (21) and Rezania *et al*. (22) established foundational protocols which demonstrated that human pluripotent stem cells could be directed to insulin producing cells that are glucose-responsive to insulin secretion, providing critical proof of concept for *in vitro* beta cell generation (21, 22). These protocols also revealed key limitations including incomplete maturation and the presence of polyhormonal cells, highlighting that successful lineage specification does not always indicate functional maturity. Subsequent work by Hogrebe *et al*. introduced refinements in temporal signaling control that improved the induction of endocrine cells, highlighting the importance of cytoskeletal differentiation cues (20), factors that were not emphasized in early differentiation protocols. Building on this, Balboa *et al*. developed an optimized multistage protocol with three-dimensional aggregation and controlled maturation steps (19), improving reproducibility and functional performance across pluripotent stem cell lines. More recently, Brassard *et al*. expanded the conceptual framework by developing a pancreatic organoid system that more faithfully recapitulate tissue architecture and morphogenesis, incorporating epithelial organization and multicellular interactions that are absent in conventional SC-islet models (23).

Despite these advances, there remain several challenges that are very relevant to disease modeling. SC-islets frequently exhibit features of functional immaturity, including elevated basal insulin secretion, reduced first-phase insulin responses, and transcriptional profiles resembling fetal beta cells. Moreover, there remains significant variability across cell lines, and protocols require extensive optimization, limiting the standardization across studies. These limitations have important implications for disease modeling of monogenic beta cell disorders. For example, developmental defects due to transcription factor mutations in MODY genes may be more readily captured than subtle defects in stimulus-secretion coupling. In congenital hyperinsulinism, hypersecretion defects may be exaggerated or confounded by baseline immaturity. Therefore, interpretation of disease phenotypes must be carefully assessed and contextualized within the maturation state of the SC-islet model.

These protocols collectively highlight a central principle that successful modeling of monogenic beta cell disorders requires not only the generation of SC-islets and insulin producing cells but also careful consideration of developmental stage, cellular context, and functional maturity. Continued refinement of differentiation stage with improved maturation, incorporation of multicellular environments, and standardization across platforms is essential for the study of monogenic beta cell diseases.

## Monogenic diabetes: neonatal diabetes and maturity onset diabetes of the young

Monogenic diabetes is a group of heterogenous disorders, caused by single gene defects that impair pancreatic beta cell development, function, or survival, that lead to dysregulated insulin secretion (5). These disorders typically present earlier than polygenic forms of diabetes and follow Mendelian inheritance patterns (25, 26). At one end of the spectrum, neonatal diabetes manifests within the first six months of life and is most commonly caused by defects in genes essential for beta cell excitability, insulin biosynthesis or cellular stress responses (8, 27, 28). In contrast, MODY generally presents later in life, from late childhood to early adulthood, and is characterized by autosomal dominant inheritance with some forms being non-insulin dependent (3, 5). MODY is most frequently caused by mutations in transcription factors or metabolic regulators that govern beta cell identity and glucose sensing. Despite these clinical distinctions, both neonatal diabetes and MODY provide complementary insight into the genetic architecture of human beta cell biology. Importantly, precise molecular diagnosis of neonatal diabetes and MODY has significant clinical implications, enabling guided therapy, accurate prognostication, and family counseling. Models of neonatal diabetes and MODY also offer unique insights into the genetic control of human beta cell development and function.

## iPSC-based modeling of monogenic diabetes

Human pluripotent SC-islets have emerged as a powerful platform to model monogenic diabetes, including MODY, by enabling the study of gene-specific effects across defined stages of beta cell development and function. A key insight from studies of many MODY-associated genes is that these genes do not solely regulate mature insulin secretion but act early in pancreatic lineage specification, thereby linking developmental defects to beta cell dysfunction. A recurring theme across various models is that transcription factor haploinsufficiency results in impaired progenitor competence, altered differentiation trajectories, and incomplete beta cell maturation, leading to reduced insulin secretion.

## Transcription factor MODY: convergent disruption of developmental networks

Studies of *HNF4A*-MODY or MODY1, using iPSC-derived SC-islets, have demonstrated that *HNF4A* plays a central role in establishing pancreatic progenitor identity and downstream endocrine commitment. Transcriptomic analyses of differentiating progenitors reveals reduced expression of key developmental regulators, including PDX1, FOXA2, GATA4, RFX6, HNF1B and SOX17 (6, 8), revealing disruption of coordinated transcriptional networks required for pancreatic specification. Moreover, these developmental defects translate into impaired beta cell maturation, reduced glucose responsiveness, and diminished insulin expression. Finally, patient specific iPSC models have also revealed mutation dependent variability in phenotype, further highlighting the utility of SC-islets for shedding light on genotype specific effects and supporting precision disease modeling (29–31).

Similarly, *HNF1A*-MODY or MODY 3 models demonstrate that disruption of *HNF1A* alters both developmental trajectories and functional programs. IPSC-derived beta cells with *HNF1A* variants exhibit aberrant differentiation trajectories and reduced expression of genes essential for glucose transport (GLUT2) and insulin secretion. Studies using patient-derived iPSCs have also demonstrated sustained expression of *HNF1A* mutations in mRNA and protein in differentiated pancreatic cells, supporting a model in which persistent mutant allele and expression contribute to defective transcriptional regulation and impaired beta cell function (33). In addition, transcriptomic studies reveal downregulation of genes including *TM4SF4, GLIS3, HNF4A*, and *UGT2B4*, reflecting the interconnected regulator network between *HNF1A* and *HNF4A*. Functionally, these defects present themselves as impaired beta cell maturation and reduced insulin secretion, consistent with progressive beta cell failure. Moreover, *HNF1A* interacts with *HNF4A*, and RNA sequencing demonstrates reduced expression of *TM4SF4, GLIS3, HNF4A* and *UGT2B4*. Mechanistically, a truncating *HNF1A* variant has been shown to impair pancreatic progenitor differentiation through antagonism of *HNF1B* function, further highlighting how disruption of the HNF transcriptional network affects early pancreatic development and downstream beta cell maturation (34).

Models of *PDX1*-MODY or MODY4 further reinforce the concept that early developmental defects can cause severe beta cell dysfunction. *PDX1* is a master regulator of pancreatic specification. Its disruption leads to impaired pancreatic progenitor formation and reduced activation of critical downstream transcription factors including RFX6, HNF1B, SOX9, and NKX6.1. IPSC-derived SC-islets with *PDX1* loss of function demonstrate reduced endocrine specification, reduced beta cell maturation, and insulin secretion (10, 35). Consistent with this developmental framework, deletion of *RFX6* in iPSC-derived systems impairs islet organoid development and survival despite preservation of PDX1^+^ and NKX6.1^+^ pancreatic progenitors, indicating that *RFX6* functions downstream of early lineage specification to regulate endocrine differentiation and beta cell viability (36). Complementary patient-derived iPSC models of Mitchell-Riley syndrome further demonstrate reduced pancreatic endoderm differentiation due to *RFX6* variants, suggesting that disruption of *RFX6* can impair both early lineage allocation and endocrine maturation depending on the genetic context (37). Similarly, *FOXA2* deficiency in human iPSC-derived models leads to aberrant beta cell development, further supporting that disruption of early endodermal and pancreatic transcriptional programs impair downstream endocrine differentiation and functional maturation (38). In contrast to models of impaired lineage specification, activating mutations in *STAT3* have been shown to cause neonatal diabetes through premature induction of pancreas differentiation, demonstrating that dysregulated timing of developmental programs, instead of failure of specification alone, can lead to beta cell dysfunction (39).

Mutations in the insulin gene (*INS*) are a well-established cause of permanent neonatal diabetes and are linked to impaired insulin biosynthesis, ER stress, and beta cell dysfunction. However, recent studies using SC-islet differentiation systems demonstrate that insulin also plays an important role in the regulation of endocrine differentiation (40). Disruption of insulin production or signaling during differentiation impairs beta cell maturation and alters key expression of transcriptional regulators, suggesting that *INS*-related monogenic diabetes reflects both developmental and functional defects. These studies expand the conceptual framework of neonatal diabetes and reinforce the concept that outputs of beta cell function can also influence developmental specification and maturation of the beta cell (40–42).

In *HNF1B*-MODY or MODY5, iPSC models highlight the importance of transcription factor dosage in the regulation of pancreatic progenitor expansion. Both patient-derived and genome-edited iPSCs demonstrate that heterozygous loss of *HNF1B* impairs endocrine progenitor proliferation and reduces co-expression of key markers including PDX1 and NKX6.1. Complete loss of *HNF1B* prevents foregut and pancreatic specification. Compensatory upregulation of multiple pancreatic transcription factors including PDX1, FOXA2, ISL1, MNX1, RFX6, GATA4, and GATA6 has been reported, suggesting the presence of critical feedback regulator networks. These findings provide the mechanistic basis for pancreatic hypoplasia and early onset diabetes that is characteristic of *HNF1B*-MODY. Extending this framework, genome editing studies in human pluripotent stem cells have demonstrated that *GATA6* haploinsufficiency impairs pancreatic development, with a modifying interaction with *GATA4* (43). Consistent with this, additional studies show that *GATA6* plays a critical role across multiple stages of development, including definitive endoderm induction, pancreatic specification, and beta cell functional maturation (44). This underscores that transcription factor dosage and cooperative regulatory networks are critical for pancreas lineage specification and may contribute to phenotypic variability in monogenic diabetes (36, 44).

## Emerging and less common MODY subtypes

Additional iPSC-based studies have begun to model rarer forms of MODY, including those associated with *KCNJ11* and *CEL* variants. These models remain less well characterized, but they demonstrate the feasibility of generating SC-islets across a wide range of genetic subtypes and reinforce the potential of this platform to capture diverse disease mechanisms (32, 49, 55).

Recent large scale genetic studies have expanded the landscape of monogenic diabetes by identifying novel genes involved in development of the pancreas and beta cell dysfunction (8). These studies highlight that beyond canonical MODY genes, a broader network of developmental regulators contributes to early onset diabetes, with overlapping phenotypes that do not fit traditional diagnostic categories. Many of these genes are implicated in pancreatic lineage specification, progenitor expansion, and endocrine differentiation, reinforcing the concept that defects in developmental programming is a central mechanism that underlies monogenic diabetes. Functional validation of these newly identified variants remains a significant challenge.

IPSC-derived SC-islet models provide a complementary and highly relevant platform to extend these genetic discoveries. These systems allow direct interrogation of how novel variants can impact lineage allocation, beta cell identity, and functional maturation. The integration of genomic discovery with stem cell based modeling has the potential to accelerate the identification of causal mechanisms and refining genotype-phenotype associations across monogenic diseases.

## Implications for disease modeling

IPSC-derived SC-islet models of MODY reveal several unifying principles that extend beyond individual gene-specific effects. Firstly, transcription factor mutations frequently disrupt early developmental programs causing impaired beta cell identity and maturation, whereas metabolic defects primarily alter stimulus secretion coupling without affecting lineage specification. These models also demonstrate that beta cell dysfunction in monogenic diabetes often originates during development, challenging traditional paradigms that focus solely on mature beta cell failure. The ability to generate patient specific and isogenic models enables precise dissection of genotype-phenotype relationships while controlling for genetic background. Beyond mechanistic insight, these systems have increasingly been leveraged for translational applications. Studies have demonstrated that gene correction in patient-derived iPSCs can restore beta cell function, and transplantation of gene-edited SC-islets into mice with diabetes can reverse hyperglycemia, providing compelling proof-of-concept for mutation specific, cell-based therapies (14). These findings position SC-islets derived from iPSCs as a platform for disease modeling and also as a foundation for precision medicine approaches aimed at correcting the underlying genetic defects in monogenic diabetes.

Despite these advances, important limitations remain including functional immaturity of SC-islet models which can confound interpretation of subtle phenotypes and variability across differentiation protocols can impact reproducibility. Addressing these challenges is essential to fully leverage iPSC-based systems for modeling monogenic diabetes and for translating these insights into clinical applications.

## Congenital hyperinsulinism

Congenital hyperinsulinism, like MODY, comprises a heterogenous group of monogenic disorders characterized by inappropriate and dysregulated insulin secretion from pancreatic beta cells, resulting in severe and persistent hypoglycemia. Congenital hyperinsulinism is the most common cause of persistent and recurrent hypoglycemia in infants and children and presents typically in the neonatal or early infancy period. The genetic architecture of HI spans defects in K_ATP_ channels, metabolic enzymes, and transcriptional regulators, reflecting multiple layers of beta cell regulation including excitability, fuel sensing, and developmental control. Beyond its clinical significance, congenital hyperinsulinism provides a unique window into human molecular and cellular mechanisms that regulate beta cell function. Moreover, iPSC-based models offer a human, genotype-specific platform to interrogate these processes and to model both functional and developmental dimensions of disease.

## Human pluripotent studies for congenital hyperinsulinism

Human pluripotent stem cell based models of congenital hyperinsulinism have provided critical mechanistic insight, particularly for K_ATP_ channel related disease. K_ATP_ channel forms of HI are the most common and well characterized genetic subtype. Early proof of concept studies demonstrated that targeted deletion of *ABCC8*, which encodes the SUR1 subunit of the K_ATP_ channel, in human embryonic stem cells resulted in insulin-producing cells with constitutive insulin secretion and impaired responsiveness to potassium-mediated depolarization (56, 57). These findings recapitulate the primary electrophysiologic defect observed in patients, where loss of K_ATP_ channel activity leads to persistent beta cell depolarization and unregulated insulin release. Importantly, pharmacologic interrogation of this model demonstrated that diazoxide was ineffective in the absence of SUR1, which is consistent with clinical diazoxide unresponsiveness in patients with *ABCC8* loss-of-function mutations (56). In contrast, agents which act independent of the K_ATP_ pathway, including somatostatin analogs and calcium channel blockers, retain the ability to suppress insulin secretion, highlighting the utility of SC-islet systems for modeling therapeutic response and resistance (56).

Subsequent studies using patient-derived or genome-edited iPSCs harboring *ABCC8* variants further refined these observations by demonstrating that SC-islets exhibit hallmark features of congenital hyperinsulinism, including elevated basal insulin secretion and impaired suppression of insulin release at low glucose concentrations (57). Functional analyses confirmed either reduced or absence of K_ATP_ channel activity, linking genotype to electrophysiological phenotype. Notably, transplantation of these SC-islets into immunodeficient mice exhibited inappropriate insulin secretion, recapitulating the hyperinsulinemic phenotype observed *in vitro* (57). Beyond defects in insulin secretion, these models also revealed increased beta cell proliferation and expression of cell cycle markers suggesting that K_ATP_ channel dysfunction not only influences beta cell excitability, but also growth dynamics (57). These findings are particularly significant given that beta cell hyperplasia is observed in diffuse forms of HI and underscores the ability of iPSC-derived models to capture functional and structural aspects of the disease.

The use of isogenic iPSC systems has further enabled accurate dissection of variant specific effects in congenital hyperinsulinism. SC-islets generated from iPSCs harboring an *ABCC8* R1420H missense variant demonstrated a mild phenotype characterized by inappropriate basal insulin secretion and partial impairment of glucose responsiveness. The phenotype described in this study was milder, reflecting some residual K_ATP_ channel activity, compared to that observed with complete *ABCC8* loss of function, highlighting the clinical heterogeneity associated with missense variants. Such studies highlight the value of iPSC-derived SC-islets in resolving genotype-phenotype relationships and emphasize that not all K_ATP_ channel defects produce equivalent functional outcomes in congenital hyperinsulinism (58).

Collectively, these studies establish that iPSC-derived human SC-islets faithfully recapitulate the core pathophysiologic features of K_ATP_ channel mediated congenital hyperinsulinism, including dysregulated insulin secretion, altered electrophysiologic behavior, and variable pharmacologic responsiveness. Broadly, these studies demonstrate that SC-islet systems provide a robust and physiologically relevant platform for modeling beta cell hypersecretion disorders in a human context.

## Expanding beyond K_ATP_ channel disease: future directions

Despite major advances in the past decade, current iPSC-based models of HI remain focused on K_ATP_ channel defects. This represents an important limitation, and iPSC-based studies should be expanded to include other genetic causes of congenital hyperinsulinism. Pathogenic variants in genes including *GLUD1, HADH, GCK, HNF4A, HK1*, and others give rise to clinically and mechanistically distinct forms of congenital hyperinsulinism, involving altered metabolic flux, transcriptional regulation, and disordered glucose sensing. Modeling other non-K_ATP_ forms using patient-derived SC-islets would enable a thorough comparison of both convergent and unique mechanisms that drive inappropriate insulin secretion. Moreover, several congenital hyperinsulinism subtypes exhibit features of developmental dysregulation with altered transcriptional activity and increased beta cell proliferation. Thus, iPSC platforms offer a unique opportunity to investigate how genetic perturbations impact not only beta cell function but also lineage specification, progenitor expansion, and maturation trajectories. Integration of these developmental and functional perspectives is essential for building a comprehensive framework of congenital hyperinsulinism pathogenesis.

Expanding the diversity of iPSC-based HI models will also facilitate the development and testing of anti-hypoglycemic agents for patients with persistent and recurrent hypoglycemia. By enabling genotype-specific drug screening and mechanistic validation, SC-islet platforms can potentially refine precision medicine approaches for patients with persistent and refractory forms of congenital hyperinsulinism.

## Conclusion

The application of patient-derived iPSCs differentiated into SC-islets has advanced the study of monogenic disorders of the pancreatic beta cell, specifically in the areas of MODY and congenital hyperinsulinism (**Table 1**). These systems provide a human, genotype-specific platform to investigate early developmental defects, impaired beta cell identity, changes in proliferation, and insulin secretion. These developmental and functional processes cannot always be interrogated using primary human tissue or animal models. Across various MODY subtypes, SC-islets have revealed how transcription factor haploinsufficiency or dysfunction perturbs endocrine lineage specification, beta cell maturation, and insulin secretion. Similarly, in congenital hyperinsulinism, iPSC-derived SC-islets recapitulate disease hallmarks including excess insulin secretion, changes in K_ATP_ channel activity, and increased beta cell proliferation. These SC-islet models lend themselves well for future investigation to evaluate the effects of novel therapies on insulin secretion as well as genetic rescue strategies.

**Table 1:** Summary of several studies involving SC-islet models in monogenic diabetes and hyperinsulinism by gene.

Monogenic diabetes		
Gene	Study	Summary
*CEL*	Pellegrini et al. (46)	This study generated iPSCs-derived beta cells from a patient with *CEL*-MODY and demonstrated glucose-stimulated insulin secretion.
*CEL* *GCK* *HNF1A HNF1B HNF4A*	Teo et al. (45)	This study used polycistronic lentiviral vector for reprogramming to derive hiPSCs from patients with *HNF4A*-, *GCK*-, *HNF1A*-, *HNF1B*-, and *CEL*-MODY that are functionally similar to hiPSCs.
*FOXA2*	Elsayed et al. (37)	This study demonstrates that *FOXA2* deficiency in iPSC derived models results in abnormal pancreas development at pancreatic progenitor stage 2, consistent with beta cell deficiency and diabetes.
*GCK*	Aquel et al. (50)	This study successfully generated iPSC lines that demonstrated pluripotency from 2 patients with variants in *GCK* (*GCK*-MODY and neonatal diabetes).
	Gao et al. (51)	This study generated an iPSC line from patient with *GCK*-MODY.
*HNF1A*	Cardenas-Diaz et al. (52)	This study used genome edited human embryonic stem cells to model *HNF1A*-MODY, showing reduced beta cell maturation and insulin secretion.
	Cujba et al. (33)	This study used human iPSCs to demonstrate that a truncating variant in *HNF1A* impairs pancreatic progenitor differentiation by antagonism of *HNF1B*.
	Griscelli et al. (44)	This study generated iPSCs from a patient with *HNF1A*-MODY that showed markers of pluripotency.
	Low et al. (53)	This study used iPSCs from a patient with *HNF1A*-MODY to show that the insulin secretory defect in *HNF1A*-MODY can be due to the effects HNF1A variants on decreased GLUT2 expression and other genes in insulin secretion.
	Yabe et al. (32)	Using patient-derived iPSCs, this study demonstrates sustained mutant expression of *HNF1A*, which leads to defective transcriptional regulation and impairs beta cell function.
*HNF1B*	El-Khairi et al. (54)	This study uses iPSC-derived pancreatic cells to delineate the role of *HNF1B* in the development of the pancreas, including effects contributing to pancreatic hypoplasia seen in *HNF1B*-MODY.
	Teo et al. (55)	This study uses iPSCs-derived pancreatic cells from a patient with HNF1B-MODY to show compensatory upregulation of multiple pancreatic transcription factors including PDX1, FOXA2, ISL1, MNX1, RFX6, GATA4, and GATA6.
	Yabe et al. (56)	This study generated iPSC-derived pancreatic beta cells from a patient with *HNF1B*-MODY and examined the *HNF1B* variant in messenger ribonucleic acids during the course of differentiation in beta cells.
*HNF4A*	Braverman-Gross et al. (29)	This study utilized iPSC-derived pancreatic cells from a patient with *HNF4A*-MODY to evaluate the effects of *HNF4A* haploinsufficiency in patients.
	Carrasco et al. (31)	This study used 3D environments (3D cell aggregation vs. 3D alginate encapsulation) for iPSC-derived beta-like cells from a patient with *HNF4A*-MODY to identify a phenotype specific to the patient’s mutation in *HNF4A.*
	Ng et al. (30)	This study leveraged iPSCS from a family with *HNF4A*-MODY to show reduced HNF4A levels, downregulated foregut genes, and upregulated hindgut genes.
*INS*	Cota et al. (39)	This study used SC-islets to demonstrate that insulin can act in an autocrine/paracrine manner to influence endocrine lineage specification and maturation.
*PDX1*	Wang et al. (34)	This study used SC-islets from 2 patients with *PDX1*-MODY to demonstrate that PDX1 is crucial for beta cell differentiation and function, along with insulin synthesis and secretion.
	Wang et al. (57)	This study used iPSCs to study the effects of *PDX1* in the early development of the pancreas, identifying key target genes and also comparing these findings to those in adult human islets.
*RFX6*	Aldous et al. (35)	This study demonstrates that deletion of *RFX6* in iPSCs impairs islet organoid development, despite normal pancreas progenitor formation.
	Trott et al. (36)	This study used patient-derived iPSC models of Mitchell-Riley syndrome to demonstrate reduced pancreatic endoderm differentiation.
*STAT3*	Saarimaki-Vire et al. (38)	This study demonstrates that activating mutations in *STAT3* result in neonatal diabetes through premature induction of pancreatic differentiation.
Congenital hyperinsulinism		
Gene	Study	Summary
*ABCC8*	Guo et al. (47)	This study generated embryonic stem cell lines with heterozygous variants in *ABBC8*, demonstrating defective K_ATP_ channels that were responsive to diazoxide.
	Lithovius et al. (48)	This study used patient-derived iPSCs (with homozygous *ABCC8* variants), providing insight into alterations in proliferation and demonstrating increased cell-cycle markers and higher fraction of proliferating cells of islets with *ABCC8* variants.
	Nair et al. (49)	This study generated immature and mature SC-islets from isogenic iPSCs with SUR1 R1420H variants, demonstrating hyperinsulinemia in homozygous SC-islets and initial hyperinsulinemia in heterozygous immature SC-islets that then progresses to lower glucose responsiveness in mature SC-islets.

A unifying insight emerging from these studies is that monogenic beta cell disorders reflect perturbations that span both development and mature function, challenging the traditional paradigms that consider these processes in isolation. However, important limitations remain, including incomplete functional maturation of SC-islets, variability across differentiation protocols, and the need for more comprehensive modeling of less well studied genetic subtypes. Addressing these limitations is essential to harness the full potential of iPSC-based systems.

Looking forward, continued refinement of SC-islet differentiation, incorporation of multicellular and organoid based platforms and expansion to a broader spectrum of genetic diseases will further enhance the utility of these models. IPSC-based SC-islet models represent a powerful and indispensable approach for dissecting disease mechanisms, allowing for precision drug testing, and will ultimately inform personalized therapeutic strategies for monogenic disorders of beta cell dysfunction.
